# Supplementary material for: Empirical methods for controlling false positives and estimating confidence in ChIP-Seq peaks
Source: BMC Bioinformatics. 2008 Dec 5;9:523. doi: 10.1186/1471-2105-9-523 (PMC2628906; doi:10.1186/1471-2105-9-523)
Supplement: Additional File 1 — A variety of html documents from the USeq web site detailing the available applications, their best usage, output file type descriptions, command line menus, etc. [file 1471-2105-9-523-S1.zip › USeqUserGuides/JavaDoc.zip!/JavaDoc/edu/utah/seq/data/package-frame.html]

edu.utah.seq.data


edu.utah.seq.data

Classes 
  
ComparatorEnrichedRegionPosition
  
ComparatorEnrichedRegionScore
  
ComparatorInfoPoint
  
ComparatorPointPosition
  
ComparatorSmoothingWindowPosition
  
ComparatorSmoothingWindowScore
  
ComparatorSmoothingWindowTwoScore
  
DividePointData
  
EnrichedRegion
  
FilterPointData
  
HeatMapMaker
  
HeatMapMakerPosNeg
  
Info
  
InfoPoint
  
Point
  
PointData
  
Simulator
  
SmoothingWindow
  
SmoothingWindowInfo
  
SubSamplePointData
